# Chemical Profile and Antioxidant Activity of *Zinnia elegans* Jacq. Fractions

**DOI:** 10.3390/molecules24162934

**Published:** 2019-08-13

**Authors:** Ana Flavia Burlec, Łukasz Pecio, Cornelia Mircea, Oana Cioancă, Andreia Corciovă, Alina Nicolescu, Wiesław Oleszek, Monica Hăncianu

**Affiliations:** 1 Department of Drug Analysis, Faculty of Pharmacy, “Grigore T. Popa” University of Medicine and Pharmacy, 16 University Street, 700115 Iasi, Romania; 2 Department of Biochemistry and Crop Quality, Institute of Soil Science and Plant Cultivation—State Research Institute, Czartoryskich 8, 24-100 Puławy, Poland; 3 Department of Pharmaceutical Biochemistry and Clinical Laboratory, Faculty of Pharmacy, “Grigore T. Popa” University of Medicine and Pharmacy, 16 University Street, 700115 Iasi, Romania; 4 Department of Pharmacognosy, Faculty of Pharmacy, “Grigore T. Popa” University of Medicine and Pharmacy, 16 University Street, 700115 Iasi, Romania; 5 Center of Organic Chemistry “C.D. Nenitescu”, Romanian Academy, Spl. Independentei 202B, 060023 Bucharest, Romania

**Keywords:** *Zinnia elegans*, Asteraceae, guanidine alkaloids, HR-QTOF/MS, lipoxygenase, metal chelation

## Abstract

*Zinnia elegans* (syn. *Zinnia violacea*) is a common ornamental plant of the Asteraceae family, widely cultivated for the impressive range of flower colors and persistent bloom. Given its uncomplicated cultivation and high adaptability to harsh landscape conditions, we investigated the potential use of *Z. elegans* as a source of valuable secondary metabolites. Preliminary classification of compounds found in a methanolic extract obtained from inflorescences of *Z. elegans* cv. Caroussel was accomplished using HR LC-MS techniques. The extract was then subjected to solid-phase extraction and separation using Sephadex LH-20 column chromatography, which resulted in several fractions further investigated for their antioxidant properties through lipoxygenase inhibition and metal chelating activity assays. Moreover, following additional purification procedures, structures of some active ingredients were established by NMR spectroscopy. The investigated fractions contained polyphenolic compounds such as chlorogenic acids and apigenin, kaempferol, and quercetin glycosides. Antioxidant assays showed that certain fractions exhibit moderate 15-LOX inhibition (Fr 2, IC_50_ = 18.98 μg/mL) and metal chelation (e.g., Fr 1-2, EC_50_ = 0.714–1.037 mg/mL) activities as compared to positive controls (20.25 μg/mL for kaempferol and 0.068 mg/mL for EDTA, respectively). For Fr 2, the 15-LOX inhibition activity seems to be related to the abundance of kaempferol glycosides. The NMR analyses revealed the presence of a kaempferol 3-*O*-glycoside, and a guanidine alkaloid previously not described in this species.

## 1. Introduction

The *Zinnia* genus, belonging to the Asteraceae family, is comprised of species grown worldwide for their ornamental role. Such species are very popular especially in North America, the origin of the genus, the center of its diversity being placed in Mexico [1,2]. *Zinnia elegans* (syn. *Z. violacea*), also known as elegant or common zinnia, is the most known and cultivated plant of this genus and was introduced in Europe around 1790, when it started gaining popularity as a garden plant [1,3].

Given its main use as an ornamental plant, very few studies focus on the analysis of secondary metabolites found in the plant in correlation with the plant’s therapeutic potential. Some investigations revealed the presence of several classes of natural compounds in certain organs of the plant. Studies of alcoholic extracts obtained from the whole plant or from leaves revealed the presence of saponins, flavonoids, polyphenols, steroids, and glycosides [4,5].

Some species of the Zinnia genus have been studied for their potential biological actions, such as antifungal [4], antioxidant, hepatoprotective [5], antibacterial, antiviral [6,7], antimalarial [8], cytotoxic (demonstrated on cancer cell lines) [9], and insecticidal [10]. However, there are few studies regarding the biological actions of extracts obtained from *Z. elegans* or of its purified compounds. Among these, research regarding the antioxidant, hepatoprotective, antifungal, and antimalarial activities can be found in the literature [4,5,8]. One of our previous studies has revealed that the methanolic extract contains important quantities of flavonoids and presents better antioxidant activity than other extracts obtained using more lipophilic solvents such as chloroform or hexane, suggesting that responsible for this type of activity are polyphenols [11].

Taking into consideration the constant need to discover new plants and implicitly new sources of secondary metabolites with therapeutic potential that could be used in the treatment of inflammatory diseases and cancer [12], we focused our attention on an ornamental plant which has been widely cultivated and in which certain classes of compounds such as polyphenols have already been identified, a fact that might suggest the existence of a therapeutic potential. Moreover, ethnopharmacological data regarding the plant in question provides the example of application of an infusion used for the treatment of pain [13]. Therefore, this study aimed to conduct a phytochemical characterization of the methanolic extract obtained from *Z. elegans* inflorescences and its fractions, as well as to evaluate the antioxidant activity through two different mechanisms (inhibition of lipoxygenase and iron chelation).

## 2. Results

### 2.1. Identification of the Constituents Found in the Z. elegans Extract

**Figure 1:**
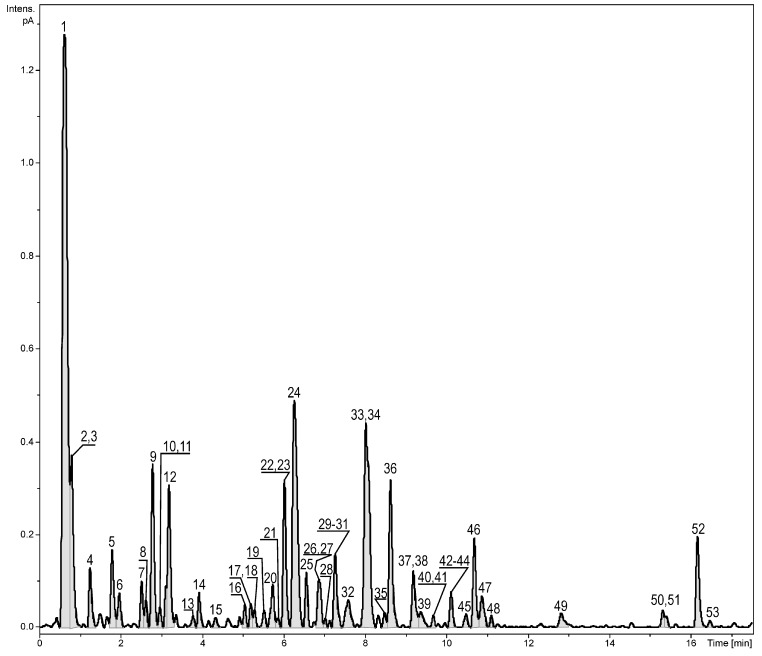
UHPLC-CAD profile of the *Z. elegans* methanolic extract.

**Figure 2:**
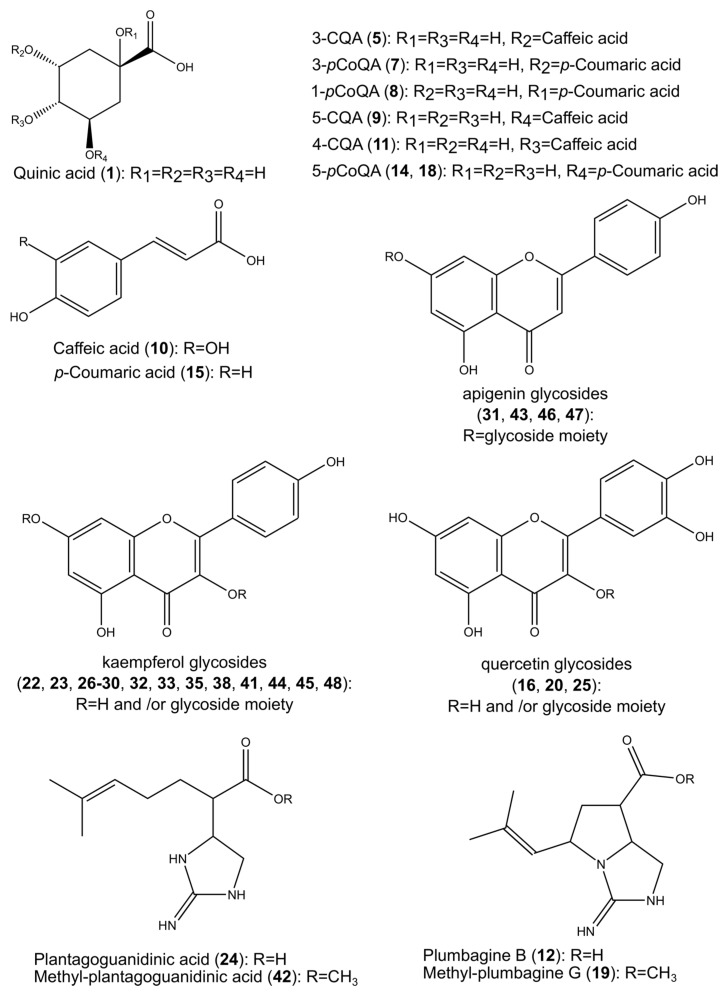
General structures and substitution patterns of phenolic acids, flavonoids, and alkaloids found in *Z*. *elegans* inflorescences.

Initial chromatographic analyses of the methanolic extract obtained from *Z. elegans* Jacq. inflorescences indicate the presence of several peaks, most of them tentatively identified as polyphenolic derivatives (Figure 1 and Table 1, peak numbers assigned based on the retention time). Over 50 compounds were tentatively identified with the help of accurate mass measurements, fragmentation patterns, retention times, UV-Vis (UltraViolet–Visible) spectra, and using the existing literature. A multistep purification procedure led to the isolation of some compounds, which were further analyzed using high-resolution mass spectrometry, one-dimensional ^1^H-, and ^13^C-NMR (Nuclear Magnetic Resonance) spectroscopy. Based on these results, we identified one new compound and a series of other metabolites already described in the literature, out of which one guanidine alkaloid (plantagoguanidinic acid) is presently reported for the first time in *Z. elegans* (Figure 2).

After further purification of the initial methanolic extract using different techniques such as solid phase extraction and LH-20 column chromatography, five fractions were obtained. The first fraction contains as main components two guanidine alkaloids (plumbagine B and plantagoguanidinic acid), while fraction 2 contains mostly monoacylchlorogenic acids such as 3-CQA, 5-CQA, 4-CQA, 3- and 5-pCoQA and also flavonoids such as kaempferol 3-*O*-β-glucopyranosyl-(1→2)]-β-glucuronopyranoside and kaempferol 3-*O*-pentoside-7-*O*-hexuronide. Fraction 3 generally contains caffeic acid, clovamide, kaempferol 3-*O*-(pentosyl-hexuronide), kaempferol 3-*O*-pentoside-7-*O*-hexuronide, resokaempferol 3-*O*-hexoside, and apigenin 7-*O*-dihexoside. Fraction 4 contains quercetin 3-*O*-hexoside, diacylchlorogenic acids such as 1,5- and 3,5-diCQA, resokaempferol 3-*O*-hexoside and apigenin 7-*O*-(malonyl-hexoside), while the last fraction (Fr 5) contains diacylchlorogenic acids, kaempferol-3-*O*-(malonyl-hexoside), kaempferol-3-*O*-hexoside, and apigenin. Compounds identified in the initial methanolic extract can be found in one or more fractions, as can be seen in Table 1.

### 2.2. Structural Characterization of the New Compounds

**Figure 3:**
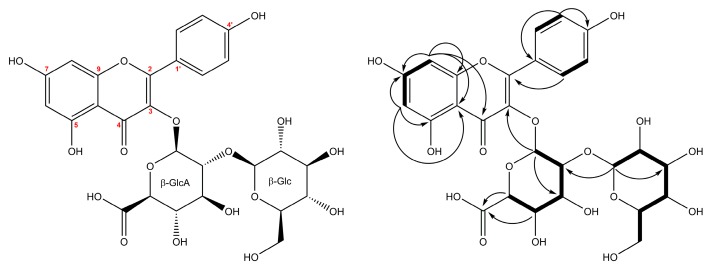
Structure of compound (**22**) isolated from the flowers of *Z. elegans* and key HMBC (H→C) and ^1^H-^1^H COSY (—) correlations.

The multistep chromatographic separation of the initial methanolic extract of *Z. elegans* flowers led to the isolation and ^1^H- and ^13^C-NMR characterization of one new kaempferol 3-*O*-glycoside (**22**) (Figure 3). Moreover, we report the presence in *Z. elegans* of one known, but rarely described guanidine alkaloid-plantagoguanidinic acid (**24**).

Compound **22** was isolated as a yellow amorphous solid with its UV spectrum presenting absorption at 265 nm (Band II) and 345 nm (Band I) [26]. The negative- and positive-ion HR-QTOF-ESI-MS spectra of **22** showed protonated and deprotonated molecule at *m*/*z* 625.1388 and *m*/*z* 623.1266, respectively. On this basis, its molecular formula was determined as C_27_H_28_O_17_. The MS/MS spectra obtained in the negative and positive ionization mode provided structural information about the type of aglycone and sequence of sugars in the oligosaccharide moiety. Namely, ion peak at *m/z* 623.1266 [M − H]^−^ gave fragment ions at *m*/*z* 285.0405 [Aglycone − H]^−^ (−338 u = C_12_H_18_O_11_ = HexA-Hex), and series of fragments belonging to kaempferol moiety at *m*/*z* 257.0455 [Aglycone − CO − H]^−^, 241.0506 [Aglycone − CO_2_ − H]^−^, 229.0506 [Aglycone − C_2_O_2_-H]^-^, 213.0557 [Aglycone − C_2_O_3_ − H]^-^ and 185.0608 [Aglycone − C_3_O_4_ − H]^−^ [25]. In the MS/MS spectrum of ion peak at *m*/*z* 625.1388 [M + H]^+^ the following fragment ions were observed: *m*/*z* 463.0870 [M − C_6_H_10_O_5_ + H]^+^ and *m*/*z* 287.0547 [M − C_12_H_18_O_11_ + H]^+^ corresponding to the kaempferol Y_0_^+^ ion, thus suggesting the 3-*O*-glycosylation of kaempferol with a disaccharide sugar chain consisting a linked hexose-hexuronic acid [27]. The analysis of ^13^C-NMR spectra of **22** showed 27 signals, sorted by the Distortionless Enhancement by Polarization Transfer with retention of Quaternaries (DEPTQ) and Heteronuclear Single Quantum Coherence (HSQC) experiments into 1 CH_2_, 16 CH and ten quaternary carbons. The aromatic region of ^1^H-NMR and 2D-COSY (COrrelation SpectroscopY) spectra of compound **22** exhibited the presence of two sets of protons due to flavonol-type aglycon. One set was attributable to a tetra-substituted aromatic ring with two *meta*-coupled protons and appeared at δ_H_ 6.39 (1H, d, *J* = 2.0 Hz, H-8) and 6.20 (1H, d, *J* = 2.0 Hz, H-6) which correlated in the HSQC spectrum with carbon atoms at δ_C_ 94.8 (C-8) and 99.9 (C-6) (Table 2.). The other set corresponded to the *para*-substituted aromatic group at δ_H_ 8.02 (1H, d, *J* = 8.5 Hz, H-2′/H-6′) and δ_H_ 6.90 (1H, d, *J* = 8.5 Hz, H-3′/H-5′), following AA’XX’ system of ring B of the aglycon. The assignments of remaining carbons of the flavonol moiety were completed by interpretation of the HMBC (Heteronuclear Multiple Bond Coherence) spectra–long-range correlations from H-2′/H-6′ to C-2 (δ 159.0) and C-4′ (δ 161.5), correlation from H-6/H-8 to C-7 (δ 165.9) and C-10 (δ 105.8), from H-6 to C-4 (δ 179.4) and C-5 (δ 163.1) and from H-8 to C-9 (δ 158.5). The carbohydrate region of ^1^H-NMR spectrum displayed the presence of two anomeric proton signals at δ_H_ 5.56 (1H, d, J = 7.4 Hz, H-1′’) and 4.75 (1H, d, *J* = 7.3 Hz, H-1′′′), indicating the presence of two sugar units. These units were elucidated as β-glucuronopyranoside δ_H/C_ 5.56 (H-1′’)/101.1 (C-1′’) and β-glucopyranoside δ_H/C_ 4.75 (H-1′’’)/104.6 (C-1′’’) based on the values of ^1^H-^1^H and ^1^J_HC_ coupling constants, and the analysis of 1D TOCSY (Total Correlation Spectroscopy) and 1D ROESY (Rotating frame nuclear Overhauser Effect SpectroscopY), HSQC, F2-coupled HSQC [28] and HMBC. The α/β-orientation of anomeric protons evidenced by the large (~7 Hz) vicinal ^1^H-^1^H coupling constants and measurements of direct ^1^H-^13^C ^1^*J* coupling constants, with values of ~170 and ~160 Hz [29], respectively, measured in F2-coupled HSQC experiment. The unusually high value of ^1^*J*_HC_ for β-GlcA was an indicator that this moiety was attached to the C-3 (δ 134.7) of the aglycon [30] and it was confirmed by the long-range correlation visible in the HMBC spectrum between H-1′’ and C-3. The ^3^J_HC_ correlation observed in the HMBC spectrum between anomeric proton of the glucose H-1′′′ and C-2′’ (δ 81.9), together with the NOE effect detected in the 1D ROESY experiment between H-1′′′ and H-2′’ (δ 3.81) indicated the presence of 1′′′→ 2′’ interglycosidic linkage. Hence, compound **22** was identified as kaempferol 3-*O*-[β-glucopyranosyl-(1→2)-β-glucuronopyranoside].

### 2.3. Antioxidant Activity

The antioxidant activity of the fractions obtained from the methanolic extract of *Z. elegans* inflorescences was determined using two well-known methods: 15-LOX inhibition assay and the iron-chelating activity test. The ability of the tested samples to chelate iron ions, as well as the capacity to inhibit lipoxygenase were expressed using EC_50_ and IC_50_ values (Table 3). The results were also compared to the values obtained for the positive controls (kaempferol and ethylenediaminetetraacetic acid—EDTA, respectively), in order to assess their efficiency.

Regarding the lipoxygenase inhibition activity, fraction 2, which contains as one of the most abundant compounds a kaempferol glycoside, presented the most promising activity (18.98 ± 0.22 μg/mL final solution), similar to that of the positive control (kaempferol). Other fractions such as Fr 3 and Fr 4 also presented a good inhibitory activity of the enzyme, while Fr 5, containing less polar compounds than the previous fractions, presented similar activity to that of the initial methanolic extract. Generally, the obtained fractions presented better IC_50_ values for the lipoxygenase inhibition assay than the total extract. On the other hand, the iron-chelating activity was most promising for the initial extract (0.615 ± 0.001 mg/mL final solution) rather than for its selective fractions. However, the calculated value was 10 times higher than that obtained for EDTA, a well-known metal chelator, implying the existence of a lower antioxidant effect explained through this mechanism. Fr 1 had the lowest EC_50_ value (0.714 ± 0.001 mg/mL final solution) out of the tested fractions.

## 3. Discussion

Several classes of natural metabolites have been previously identified in *Z. elegans*. Among the flavonoids previously identified in the plant were apigenin 7-*O*-glucoside, apigenin 4′-*O*-glucoside, kaempferol 3-*O*-glucoside, kaempferol 3-*O*-xyloside-7-*O*-glucoside, luteolin 7-*O*-glucoside, and quercetin 3-*O*-glucoside [6,31]. Moreover, acetylated cyanidin and pelargonidin diglucosides have also been reported in the inflorescences of *Z. elegans* [6,32].

Another class of metabolites confirmed for the species in question is that of terpenoids, which can be found in the volatile oil, as well as in certain organic extracts. Sesquiterpenes such as ziniolide, germacren D, zinaflorin III and other related compounds have been identified in extracts obtained from the aerial parts or roots [6,33]. The volatile oil obtained from inflorescences presented germacren D and *p*-cymene as major constituents [34].

A screening of new plants containing oil with potential industrial applications revealed that the seeds harvested from the plant contain an important amount of oil (28%) [35]. The content of saturated fatty acids was found to be 29%, while monounsaturated fatty acids were present in a higher proportion (48%) [35,36]. Moreover, *Z. elegans* was also reported to contain acetylenic compounds [37], as well as nicotine-derived alkaloids [38].

In a recent study focusing on the analysis of secondary metabolites found in an ethanolic extract of *Z. elegans* and its fractions through LC-MS techniques, two coumarins (esculetin and umbelliferone), two sesquiterpene lactones (zaluzanin C and 8β-(angeloyloxy)-1β-hydroxyarbusculin B), and some phenylethanoids such as acteoside were identified [8].

The present chemical analysis of the methanolic extract revealed the presence of numerous polyphenolic compounds such as monoacyl- and diacylchlorogenic acids and glycosides of kaempferol, apigenin, quercetin, and resokaempferol, as well as of several amino acids and guanidine alkaloids. To the best of our knowledge, plantagoguanidinic acid (isolated from Fr 1) and kaempferol 3-*O*-[β-glucopyranosyl-(1→2)-β-glucuronopyranoside] (isolated from Fr 2) have not been previously reported in *Z. elegans* extracts. Moreover, the latter kaempferol glycoside is being described for the first time in literature in the present work.

The five LH-20 fractions (Fr 1–5) obtained after the purification of the 85% MeOH fraction contain mostly polyphenolic compounds, except for the first fraction containing mostly guanidine alkaloids. It can be observed that starting with Fr 1, the polarity of the eluted compounds starts to decrease. Therefore, the last fraction contains more nonpolar compounds such as apigenin, while the middle fractions contain mostly flavonoid glycosides. As expected, flavonoids containing more sugar moieties were some of the first eluted compounds, while flavonoids with only one sugar group and aglycons can be found towards the end of the separation.

Recently, more and more research regarding the antioxidant activity of medicinal plants has been conducted to discover new plant metabolites that could be used in the treatment of diseases associated with oxidative processes and inflammation, such as cancer and cardiovascular diseases [39,40]. Probably the most important and widespread class of natural antioxidants are polyphenols. Compounds from this class are utilized for their potential beneficial effects in the prevention of various diseases [41]. Several mechanisms of action can explain the antioxidant activity of polyphenolic compounds, but the most common ones are the radical scavenging activity, metal chelation, and inhibition of enzymes involved in the production of free radicals [41,42].

The current study correlates the presence of certain polyphenols such as polycarboxylic acids and flavonoids from *Z. elegans* inflorescences to the antioxidant activity observed through pro-inflammatory enzyme inhibition and metal chelation mechanisms. 

Lipoxygenases are a family of enzymes involved in the oxidation of polyunsaturated fatty acids and have different physiological roles, as well as implications in several pathological processes [43]. 15-LOX, one of the enzymes belonging to this group, has recently attracted attention due to its connection to largely spread diseases such as cancer, Alzheimer’s disease, and diabetes, a fact that has led to the set-up of a new study direction involving the research for the discovery of new potent 15-LOX inhibitors [44]. Previous studies have shown that polyphenols can act as LOX inhibitors and are responsible for the protective effect against inflammation and oxidation [45,46]. Our results suggest that such compounds have good antioxidant and anti-inflammatory activities given the observed inhibition of the enzyme. It can be noted that purified fractions (Fr 2–4) containing polyphenols such as chlorogenic acids and kaempferol, apigenin and quercetin glycosides present a better inhibitory activity than the total extract, which suggests that other compounds present in it might reduce its ability to inhibit the enzyme. Therefore, the obtained values also indicate that certain polyphenol-rich fractions can present improved antioxidant and anti-inflammatory activities compared to the crude extracts, which justifies the current trend in pharmacognostic research regarding the importance of separation and purification of compounds from total extracts.

Biochemical reactions leading to the production of reactive oxygen species (ROS) are dependent on the presence of several metal ions, such as iron and copper, that act as catalyzers or are directly involved in ROS synthesis [47,48]. Under certain conditions, higher production of ROS can generate oxidative stress, leading to the deterioration of several cell structures and modification of certain substances (e.g., nucleic acids), with severe consequences for hemostasis. Therefore, the chelation of such reduced metals leads to a reduction in the formation of ROS by lowering the available amount of catalyst. This represents one of the possible mechanisms through which substances with certain functional groups such as hydroxyl, carbonyl, and amino can act as antioxidant molecules [48].

The results regarding the metal chelation assay are rather different from the ones obtained for the LOX inhibition, suggesting that the total extract has a better capacity of chelating iron ions than the more purified fractions. Although flavonoids such as kaempferol and quercetin are known as good metal chelators, the values could be explained by the presence of more polyphenolic compounds (e.g., tannins), and implicitly, more hydroxyl groups in the initial extract that can block iron ions [48,49,50]. Fr 1, which contains mostly alkaloids such as plantagoguanidinic acid, appears to have the best metal-chelating activity out of the tested fractions. Although polyphenols are the most tested compounds for their metal-chelating activity, it has also been demonstrated that other compounds such as alkaloids presenting at least a free nitrogen atom also exhibit iron-binding capacity [51]. However, Fr 1 presented one of the highest IC_50_ in the lipoxygenase inhibition assay, which implies a rather weak antioxidant activity. Nevertheless, lipoxygenase inhibition is a more complex process, involving different mechanisms and compounds with such inhibitory properties can either act upon the active site of the enzyme by reducing the ferric ion to its ferrous form, by blocking the ferrous form, or they can alter its tridimensional structure, consequently reducing or blocking its activity [52]. Therefore, the existence of possible synergistic effects of plant constituents could explain why sometimes plant extracts are more active than a specific natural compound.

## 4. Materials and Methods

### 4.1. Chemicals and Reagents

LC-MS grade acetonitrile and HPLC grade methanol were purchased from Merck (Darmstadt, Germany). MS-grade formic acid was purchased from Sigma Aldrich (Steinheim, Germany) and ultrapure water was obtained using a Milli-Q Simplicity 185 water purification system (Millipore, Milford, MA, USA). For biochemical tests, lipoxidase from *Glycine max* (soybean) type I-B, as well as linoleic acid, kaempferol (analytical grade) and EDTA were purchased from Sigma Aldrich (Steinheim, Germany), while the acetate buffer 0.1 M pH = 5.25 was prepared by mixing sodium acetate 0.1 M solution with acetic acid (Sigma Aldrich) until the appropriate value of pH was obtained. Similarly, borate buffer (pH 9) was obtained by mixing boric acid (Sigma Aldrich) with NaOH 1 N until the appropriate value of pH was reached. Moreover, the ferrous sulfate solution in 0.2 M hydrochloric acid and the 5 mM ferrozine solution were also obtained by dissolution using the appropriate chemicals and reagents acquired from Sigma Aldrich (Steinheim, Germany).

### 4.2. Plant Material

*Zinnia elegans* cv. Caroussel was cultivated in ecological conditions in the north-eastern part of Romania in the year 2017. Inflorescences were harvested and kept in the Pharmacognosy department of *Grigore T. Popa* University of Medicine and Pharmacy Iași, being assigned the voucher specimen code Zf 2017. The inflorescences were ground using a commercial blender. Ten grams of the obtained powder was weighed, and afterward 200 mL of methanol was added. The extraction was conducted using a magnetic stirrer (DLAB MS-M-S10, Beijing, China) for 3 hours at room temperature. The extract was then filtered through filter paper, and the solvent was evaporated to dryness in a rotary evaporator (150 mbar pressure, temperature 40 °C). The obtained extract was stored at 4 °C until further use.

### 4.3. Isolation

The crude methanol extract was purified with the help of various chromatographic methods. Firstly, the extract was subjected to solid phase extraction using a preconditioned RP-C_18_ column (100 × 80 mm i.d.; Cosmosil 140C_18_-PREP, 140 µm), followed by removal of compounds with high polarity (1% MeOH *v*/*v*, 50% MeOH *v*/*v*), while a phenolic-rich fraction was eluted with a solution containing 85% methanol and 0.1% formic acid.

The 85% methanol fraction was further purified on a Sephadex LH-20 (Sigma-Aldrich, Steinheim, Germany) column (970 × 34 mm i.d.) and eluted with MeOH 100%. As a result of this separation, 5 fractions (Fr 1–5) were collected. The composition of the fractions was monitored by LC-MS techniques. After further purification using a semi-preparative HPLC chromatographic system, two compounds that have not been previously reported in *Z. elegans* were obtained from fractions Fr 1 and Fr 2, respectively.

### 4.4. Semi-Preparative HPLC

Further purification of two LH-20 fractions involved the use of a semi-preparative HPLC Gilson chromatographic system (Gilson Inc., Middleton, WI, USA), equipped with an evaporative light scattering detector (ESLD, Gilson PrepELS II). This purification step was achieved using a RP-C_18_ Kromasil 100-5-C18 column (250 × 10 mm i.d.; 5 µm). The separation was carried out in gradient mode, using aqueous acetonitrile solution (10–60% *v*/*v*), containing 0.1% formic acid. The column was maintained at 40 °C, and the mobile phase flow rate was 4 mL/min.

### 4.5. High-Resolution LC-MS and Qualitative Analysis

The crude methanolic extract, as well as LH-20 fractions, were subjected to high-resolution LC-MS analyses. Chromatographic separations were carried out using Thermo Scientific Ultimate 3000RS chromatographic system on a Waters BEH C18 column (150 × 2.1 mm i.d.; 1.7 µm, Milford, USA) held at 50 °C. The separation of the compounds of interest was achieved using concave-shaped gradient (Dionex gradient curve nr. 6) from 5% to 60% of phase B (acetonitrile containing 0.1% formic acid) in phase A (0.1% formic acid in distilled water) over 25 min. The flow rate was 0.55 mL/min. Between the injections, the column was equilibrated with ten volumes of 5% phase B.

The column effluent passed through the flow cell of photodiode array detector, recording absorbances in the 200–600 nm wavelength range with 5 nm bandwidth and 10 Hz acquisition frequency. A flow splitter was then used to divert the column effluent in 1:3 proportion between Q-TOF MS (Bruker Impact II HD, Bruker, Billerica, MA, USA) and charged aerosol detector (CAD, Thermo Corona Veo RS) connected in parallel. CAD acquisition frequency was 10 Hz.

The mass spectrometric analyses were carried out in both positive and negative ion mode with electrospray ionization. Linear (centroid) spectra were acquired over a mass range from *m/z* 50 to *m/z* 2000 at 5 Hz acquisition frequency with the following parameters of mass spectrometer: positive ion capillary voltage 4.5 kV; negative ion capillary voltage 3.0 kV, dry gas flow 6 L/min; dry gas temperature 200° C; nebulizer pressure 0.7 bar; collision cell transfer time 90 μs; prepulse storage 7.0 μs. In each scan, two precursor ions with intensities of over 2000 counts were fragmented. The collision energy was set automatically depending on the *m*/*z* of fragmented ion, in the range of 5 to 100 eV. Acquired data were calibrated internally with sodium formate introduced into the ion source via a 20 µL loop at the beginning of each separation. Data acquisition and processing was performed using Bruker DataAnalysis 4.3 software.

### 4.6. NMR Spectroscopy

The 1D- and 2D-NMR spectra (^1^H, ^13^C DEPTQ, ^1^H-^13^C HSQC, ^1^H-^13^C H2BC, ^1^H-^13^C HMBC, ^1^H-^13^C F2-coupled HSQC, ^1^H-^1^H COSY, 1D-TOCSY, 1D-ROESY) were acquired using an Avance III HD Ascend 500 MHz spectrometer (Bruker BioSpin, Rheinstetten, Germany), in MeOH-*d_4_* with 0.1% of trifluoroacetic acid at 30 °C.

#### Characteristic Data of Isolated Z. elegans Compounds

Kaempferol 3-*O*-[β-glucopyranosyl-(1→2)-β-glucuronopyranoside] (**22**); yellow amorphous solid; UV (PDA, MeCN/H_2_O) λ_max_ (nm) 265, 345; HR-QTOF-MS (neg.) *m/z* 623.1266 [M − H]^−^ (calc. for C_27_H_27_O_17_ 623.1254). ^1^H- and ^13^C-NMR spectroscopic data (Table 2).

Plantagoguanidinic acid (**24**); colorless oil; HR-QTOF-MS (neg.) *m*/*z* 224.1408 [M − H]^−^ (calc. for C_11_H_18_N_3_O_2_ 224.1405). ^13^C-NMR (125 MHz, MeOH-*d_4_*) δ 181.0 (C-1), 161.3 (C-2′), 133.2 (C-6), 124.9 (C-5), 58.5 (C-4′), 53.0 (C-2), 48.0 (C-5′), 30.0 (C-3), 26.9 (C-4), 25.9 (C-8), 17.8 (C-7); ^1^H-NMR (500 MHz, MeOH-*d_4_*) δ 2.45 (1H, m, H-2), 1.64 (1H, m, H-3a), 1.57 (1H, m, H-3b), 2.27 (1H, m, H-4a), 2.10 (1H, m, H-4b), 5.14 (1H, t, *J* = 6.9 Hz, H-5), 1.61 (3H, s, H-7), 1.68 (3H, s, H-8), 4.16 (1H, m, H-4′), 3.76 (1H, t, *J* = 9.5 Hz, H-5′a), 3.54 (1H, m, H-5′b).

^1^H- and ^13^C-NMR spectra of these compounds are available in the Supplementary Materials.

### 4.7. Antioxidant Tests

#### 4.7.1. Lipoxygenase Inhibition

The lipoxygenase inhibition activity was evaluated using the amended Malterud method [53]. 0.05 mL of lipoxidase from *Glycine max* (soybean) in borate buffer (pH 9) was mixed with the same volume of the sample solution in DMSO (in various concentrations). After 10 minutes, 2 mL of 0.16 mM linoleic acid borate buffer were added and the absorbances were registered at 234 nm for 90 seconds. The inhibition of lipoxygenase was established using the following formula: % inhibition = (A_EFI_ − A_ECI_) × 100/A_EFI_; A_EFI_ is the difference of the enzyme absorbance without inhibitor at 90 and 30 seconds, while A_ECI_ represents the same difference of the enzyme-inhibitor mixture. Kaempferol was used as positive control and the IC_50_ values were calculated for each sample and expressed as µg/mL. All experiments were performed in triplicate.

#### 4.7.2. Metal Chelation

The potential to chelate ferrous ions was determined for the investigated extracts according to the method described by Venditti et al. with some modifications [54,55]. The ferrous ions form with ferrozine a complex with maximum absorbance at 562 nm. Consequently, the presence of a chelating agent in the reaction medium decreases the absorbance of the complex. 0.2 mL sample solution, 0.74 mL 0.1 M acetate buffer (pH 5.25) and 0.02 mL 2 mM ferrous sulphate solution in 0.2 M hydrochloric acid were mixed. After 10–15 s, 0.04 mL of 5 mM ferrozine solution was added. The absorbance of the solution was determined after being kept for 10 min in the dark, against a blank prepared under similar conditions. The metal chelating activity was determined using the following formula: Activity % = 100 × (Ac − Ap)/(Ac), where Ac is the absorbance of the control solution and Ap is the absorbance of the sample solution. EDTA was used as positive control. The EC_50_ was calculated for each extract and expressed as mg sample/mL final solution. The assay was carried out in triplicate.

#### 4.7.3. Statistical Analysis

The one-way ANOVA followed by Tukey’s honest significant difference test was performed using freely available web-based online software https://houssein-assaad.shinyapps.io/SumAOV/ [56]. The chosen level of significance was *p* < 0.05. Data are expressed as means ± standard deviation.

## 5. Conclusions

This study focused on a phytochemical HR LC-MS analysis of a methanolic extract obtained from *Z. elegans* inflorescences, in which more than 50 compounds from different classes, such as polyphenols and alkaloids, were identified. After further separations, five fractions were chemically characterized and tested for potential antioxidant activities. Fractions Fr 2 with a rich content in monoacylchlorogenic acids and flavonoid glycosides and Fr 1, having alkaloids as major constituents, showed promising results. Therefore, these two fractions were subjected to additional purification, and two compounds (plantagoguanidinic acid and a new kaempferol glycoside), which have not been previously reported in *Z. elegans,* were isolated and characterized using NMR techniques. In conclusion, this paper is part of current scientific trends; namely, the discovery of new sources of natural metabolites with biological actions and the isolation of such compounds for potential therapeutic applications.

## Figures and Tables

**Table 1:** Compounds identified in the *Z. elegans* methanolic extract and the obtained fractions using UHPLC-QTOF-MS/MS.

No.	Compound Name	RT (min)	λ_max_ (nm)	Formula	Error (ppm) **	mσ ***	Observed [M − H]^−^	Major Fragments (%)	Fraction	Reference
1.	Quinic acid	0.62	-	C_7_H_12_O_6_	0.5	1.5	191.0560	173.0450 (2)	1,2	[14]
2.	Hexoso(iso)leucine	0.79	-	C_12_H_23_NO_7_	−0.7	1.4	294.1549 *	276.1443 (100), 258.1338 (19), 230.1388 (17), 294.1548 (12), 132.1021 (5)	1	[15]
3.	(Iso)leucine	0.79	-	C_6_H_13_NO_2_	−0.6	2.8	132.1020 *	132.1019 (100), 86.0967 (1)	1	[15]
4.	Phenylalanine	1.24	-	C_9_H_11_NO_2_	5.3	15.7	164.0708	-	1	[15]
5.	3-CQA	1.79	250, 325	C_16_H_18_O_9_	−0.1	3.6	353.0878	191.0565 (100), 179.0354 (37), 135.0436 (16)	1,2	[16]
6.	Tryptophan	1.96	280	C_11_H_12_N_2_O_2_	−0.9	9.1	205.0972 *	188.0708 (100), 146.0603 (26), 144.0811 (6), 205.0974 (6)	1,2	[15]
7.	3-*p*CoQA	2.51	310	C_16_H_18_O_8_	−0.4	9.0	337.0930	163.0399 (100), 119.0495 (37), 191.0556 (19)	1,2	[16]
8.	1-*p*CoQA	2.62	305	C_16_H_18_O_8_	−1.0	17.1	337.0932	191.0567 (100), 163.0400 (34), 119.0492 (9)	1,2	[16]
9.	5-CQA	2.78	245, 325	C_16_H_18_O_9_	−0.4	3.4	353.0880	191.0563 (100), 179.0265 (1), 135.0433 (1), 173.0438 (1)	1,2,3	[16]
10.	Caffeic acid	2.96	320	C_9_H_8_O_4_	−0.3	1.8	179.0353	135.0441 (100), 179.0359 (42)	2,3,4	[17]
11.	4-CQA	3.11	325	C_16_H_18_O_9_	−0.7	17.0	353.0880	191.0567 (100), 179.0351 (48), 173.0454 (40), 135.0439 (31)	2,3	[16]
12.	Plumbagine B	3.18	-	C_11_H_17_N_3_O_2_	−0.3	1.1	222.1251	222.1251 (100), 178.1351 (12)	1	[18]
13.	Plumbagine B - isomer	3.77	-	C_11_H_17_N_3_O_2_	−0.4	6.0	224.1395 *	-	1	[18]
14.	5-*p*CoQA epimer	3.92	310	C_16_H_18_O_8_	−2.5	2.0	337.0937	191.0567 (100), 163.0402 (8)	1,2	[16]
15.	*p*-Coumaric acid	4.32	310	C_9_H_8_O_3_	0.0	3.6	165.0546 *	147.0441 (100), 119.0491 (11), 165.0543 (9)	2	[17]
16.	Quercetin-3-*O*-(hexosyl-hexuronide)	5.05	255, 345	C_27_H_28_O_18_	0.8	5.6	641.1343 *	303.0499 (100), 479.0821 (40), 301.0344 (12)	2,3,4	[19]
17.	Clovamide	5.19	290, 320	C_18_H_17_NO_7_	−1.9	40.4	358.0939	178.0498 (100), 179.0346 (61), 161.0247 (30)	2,3,4	[20,21]
18.	5-*p*CoQA epimer	5.29	300	C_16_H_18_O_8_	−2.6	26.4	337.0938	191.0566 (100)	1,2	[16]
19.	Methyl-Plumbagine B	5.52	-	C_12_H_19_N_3_O_2_	1.9	10.1	238.1550 *	238.1546 (100), 178.1333 (1), 196.1326 (0.3), 136.1117 (0.2), 110.0959 (0.1)	1,2	[18]
20.	Quercetin-3-*O*-(pentosyl-hexoside)	5.73	265, 350	C_26_H_28_O_16_	−2.0	5.6	595.1316	300.0288 (100), 271.0248 (30)	2,3,4	[21]
21.	Plantagoguanidinic acid isomer	5.87	-	C_11_H_19_N_3_O_2_	2.2	9.6	226.1550 *	226.1544 (100), 208.1437 (5), 180.1488 (1), 149.0955 (1)	1	[18]
22.	Kaempferol 3-*O*-[β-glucopyranosyl-(1→2)-β-glucuronopyranoside]	6.02	265, 345	C_27_H_28_O_17_	−1.9	3.9	623.1266	285.0409 (100), 229.0510 (21), 257.0461 (15), 241.0508 (3)	2,3	-
23.	Kaempferol 3-*O*-(hexosyl-hexoside)	6.06	260, 345	C_27_H_30_O_16_	3.4	24.7	611.1586 *	287.0545 (100), 449.1073 (92), 611.1597 (38), 226.1533 (4)	3	[22]
24.	Plantagoguanidinic acid	6.26	-	C_11_H_19_N_3_O_2_	−1.4	5.1	224.1408	141.0913 (100), 224.1404 (44), 180.1510 (32)	1	[18]
25.	Quercetin 3-*O*-hexoside	6.56	255, 350	C_21_H_20_O_12_	−1.3	1.9	463.0888	300.0285 (100)	3,4,5	[23]
26.	Kaempferol 3-*O*-(pentosyl-hexoside)	6.87	265, 345	C_26_H_28_O_15_	2.1	14.3	581.1501 *	287.0542 (100), 449.1067 (9), 163.0601 (1), 145.0495 (1)	2,3,4	[19]
27.	Kaempferol 3-*O*-(pentosyl-hexuronide)	6.87	265, 345	C_26_H_26_O_16_	2.4	19.4	595.1279 *	287.0546 (100), 463.0866 (42), 273.0748 (12)	2,3,4	[19]
28.	Kaempferol 3-*O*-(pentosyl-hexoside)	7.02	265, 345	C_26_H_28_O_15_	-1.8	7.5	579.1355	284.0330 (100), 255.0311 (37), 227.0344 (17)	2,3,4	[19]
29.	Kaempferol 3-*O*-pentoside-7-*O*-hexuronide	7.27	265, 345	C_26_H_26_O_16_	1.0	13.1	595.1288 *	287.0541 (100), 463.0866 (33), 433.1130 (12), 271.0587 (10)	2,3,4	[19]
30.	Kaempferol 3-*O*-hexoside	7.27	265, 345	C_21_H_20_O_11_	2.1	3.5	449.1069 *	287.0545 (100), 449.1060 (15)	4,5	[19]
31.	Apigenin 7-*O*-dihexoside	7.27	265, 345	C_27_H_30_O_15_	−0.7	8.4	593.1516	269.0462 (100)	2,3	[19]
32.	Kaempferol 3-*O*-hexoside	7.59	260, 335	C_21_H_20_O_11_	−0.9	11.3	447.0937	284.0340 (100), 255.0312 (55)	2,3,4,5	[22]
33.	Kaempferol-3-*O*-hexuronide	8.02	245, 325	C_21_H_18_O_12_	−0.9	13.0	461.0725	285.0409 (100), 229.0502 (22), 257.0458 (10)	1,2,3,4	[19]
34.	1,5-diCQA/3,5-diCQA	8.02	245, 325	C_25_H_24_O_12_	0.1	1.7	515.1195	191.0562 (100), 179.0349 (10), 173.0450 (3)/191.0564 (100), 179.0349 (34), 135.0445 (16)353.0876 (12)	3,4,5	[16]
35.	Kaempferol-3-*O*-hexoside	8.47	265, 340	C_21_H_20_O_11_	0.1	2.7	447.0932	285.0404 (100), 257.0456 (1), 241.0497 (1)	2,3,4,5	[19]
36.	Resokaempferol 3-*O*-hexoside	8.63	265, 335	C_21_H_20_O_10_	0.6	5	431.0981	268.0379 (100)	3,4,5	[19]
37.	3,4-diCQA	9.19	325	C_25_H_24_O_12_	0.7	5.8	515.1191	191.0558 (100), 173.0451 (77)	3,4,5	[16]
38.	Kaempferol-3-*O*-(malonyl-hexoside)	9.19	325	C_24_H_22_O_14_	1.1	21.7	535.1076 *	287.0545 (100), 535.1076 (60), 285.0388 (12), 257.0442 (6), 449.1067 (3)	3,4,5	[19]
39.	*p*Co,CQA isomer	9.37	320	C_25_H_24_O_11_	1.9	8.6	501.1382 *	163.0383 (100), 147.0436 (99), 483.1278 (32), 337.0924 (5)	3	[16]
40.	C_13_-norisoprenoid hexoside	9.68	-	C_19_H_32_O_7_	2.6	14.9	373.2211 *	211.1690 (100), 193.1585 (25), 135.1162 (12), 175.1473 (11)	1	[24]
41.	Kaempferol 3-*O*-(caffeoyl-pentoside)-7-*O*-hexuronide	9.68	330	C_35_H_32_O_19_	2.4	12.7	757.1592 *	287.0546 (100), 277.0704 (36), 463.0868 (35), 163.0388 (22), 295.0807 (11)	5	[19]
42.	Methyl-plantagoguanidinic acid	10.11	-	C_12_H_21_N_3_O_2_	2.2	4.1	240.1701 *	240.1704 (100), 208.1439 (4), 181.1214 (0.6)	1	[18]
43.	Apigenin 7-*O*-(malonyl-hexoside)	10.11	330	C_24_H_22_O_13_	2.0	9.6	519.1123 *	271.0596 (100), 519.1127 (27)	3,4	[19]
44.	Kaempferol-3-*O*-(acetyl-hexoside)	10.11	330	C_23_H_22_O_12_	1.7	54.6	491.1176 *	287.0544 (100)	3	[19]
45.	Kaempferol-3-*O*-(malonyl-hexoside)	10.48	265, 330	C_24_H_22_O_14_	2.0	7.0	535.1072 *	287.0550 (100), 535.1076 (91)	3,4,5	[19]
46.	Apigenin 7-*O*-(malonyl-hexoside)	10.69	265, 335	C_24_H_22_O_13_	1.3	4.7	519.1126 *	271.0601 (100), 519.1133 (54)	3,4	[22]
47.	Apigenin 7-*O*-(malonyl-hexoside)	10.87	270, 330	C_24_H_22_O_13_	1.4	3.4	519.1126 *	519.1129 (100), 271.0599 (93)	3,4	[19]
48.	Kaempferol 3-*O*-(p-coumaroyl-pentoside)-7-*O*-hexuronide	11.10	265, 320	C_35_H_32_O_18_	1.0	27.3	741.1654 *	287.0550 (100), 261.0761 (53), 463.0873 (35), 147.0441 (21), 279.0862 (16)	3,4	[19]
49.	Apigenin	12.82	265, 335	C_15_H_10_O_5_	1.1	0.8	269.0452	225.0559 (6), 117.0326 (2)	3,4,5	[25]
50.	Di-*p*-coumaroyl-caffeoyl-C_18_H_36_O_6_	15.31	295, 310	C_45_H_54_O_13_	2.4	23.6	803.3618 *	147.0441 (100), 657.3271 (44), 163.0388 (27), 641.3336 (14), 275.1751 (13), 204.1017 (11), 511.2924 (11), 495.2958 (10), 119.0490 (8), 291.1697 (6), 655.3094 (5)	2,3	-
51.	Di-*p*-coumaroyl-caffeoyl-C_18_H_36_O_6_	15.40	295, 310	C_45_H_54_O_13_	1.2	31.6	803.3628 *	147.0441 (100), 657.3277 (47), 163.0388 (41), 204.1016 (22), 275.1750 (20), 641.3322 (20), 495.2967 (16), 511.2907 (9)	2,3	-
52.	Tri-*p*-coumaroyl-C_18_H_36_O_6_	16.17	300	C_45_H_54_O_12_	0.9	21.3	787.3681 *	147.043 (100), 641.3324 (53), 204.1017 (20), 275.1750 (15), 495.2962 (15), 119.0487 (7), 477.2854 (4), 349.2598 (1)	2,3	-
53.	3-O-Methyl-kaempferol	16.47	-	C_16_H_12_O_6_	1.0	8.9	301.0704 *	301.0704 (100)	5	[19]

* Measured in positive ESI ionization mode as [M + H]^+^. ** Mass accuracy measurements expressed in parts per million (ppm). *** Isotopic pattern fit factor (mσ).

**Table 2:** ^1^H- and ^13^C-NMR data (MeOH-*d_4_* + 0.1% trifluoroacetic acid, 500/125 MHz, 30 °C) for compound **22**.

Position.	δ_H_ (*J* in Hz)	δ_C_, Type
2		159.0, C
3		134.7, C
4		179.4, C
5		163.1, C
6	6.20 d (2.0)	99.9, CH
7		165.9, C
8	6.39 d (2.0)	94.8, CH
9		158.5, C
10		105.8, C
1′		122.7, C
2′/6′	8.02 d (8.5)	132.3, CH
3′/5′	6.90 d (8.5)	116.3, CH
4′		161.5, C
1′’	5.56 d (7.4)	101.1, CH
2′’	3.81 dd (9.0, 7.4)	81.9, CH
3′’	3.67 t (9.0)	77.3, CH
4′’	3.64 t (9.0)	72.6, CH
5′’	3.76 d (9.0)	76.8, CH
6′’		171.7, C
1′’’	4.75 d (7.3)	104.6, CH
2′’’	3.35 dd (9.5, 7.3)	75.5, CH
3′’’	3.38 overlap	77.9, CH
4′’’	3.37 overlap	71.3, CH
5′’’	3.28 ddd (8.4, 5.0, 2.2)	78.2, CH
6′’’	3.78 dd (12.0, 2.2)3.68 dd (12.0, 5.0)	62.6, CH_2_

**Table 3:** Antioxidant activities of *Z. elegans* fractions.

	Lipoxygenase Inhibition	Iron-Chelating Activity
Sample	IC_50_ (μg/mL Final Solution)	EC_50_ (mg/mL Final Solution)
Fr 1	65.65 ± 0.50 ^a^ *	0.714 ± 0.001 ^e^
Fr 2	18.98 ± 0.22 ^d^	1.037 ± 0.003 ^d^
Fr 3	30.25 ± 0.73 ^c^	1.620 ± 0.006 ^c^
Fr 4	41.67 ± 1.46 ^b^	1.919 ± 0.011 ^a^
Fr 5	69.37 ± 6.71 ^a^	1.664 ± 0.011 ^b^
Initial extract	69.21 ± 0.89 ** ^a^	0.615 ± 0.001 ^f^
Positive control	20.25 ± 0.44 ^d^	0.068 ± 0.003 ^g^

* Values are the means ± standard deviation, *n* = 3. ^a–g^ Means in a column without a common superscript letter differ (*p* < 0.05), as indicated by one-way ANOVA. ** Data already published [11].

## Supplementary Materials

The supplementary materials are available online.
