# Supplementary material for: Chemical Profile and Antioxidant Activity of Zinnia elegans Jacq. Fractions
Source: Molecules. 2019 Aug 13;24(16):2934. doi: 10.3390/molecules24162934 (PMC6720917; doi:10.3390/molecules24162934)

Supplementary Materials

Chemical Profile and Antioxidant Activity of *Zinnia elegans* Jacq. Fractions

Ana Flavia Burlec [1,†], Łukasz Pecio [2,†], Cornelia Mircea [3,*] Oana Cioancă [4], Andreia Corciovă [1,*], Alina Nicolescu [5], Wiesław Oleszek [2] and Monica Hăncianu [4]

[1] Department of Drug Analysis, Faculty of Pharmacy, "Grigore T. Popa" University of Medicine and Pharmacy, 16 University Street, 700115 Iasi, Romania
[2] Department of Biochemistry and Crop Quality, Institute of Soil Science and Plant Cultivation—State Research Institute, Czartoryskich 8, 24-100 Puławy, Poland
[3] Department of Pharmaceutical Biochemistry and Clinical Laboratory, Faculty of Pharmacy, "Grigore T. Popa" University of Medicine and Pharmacy, 16 University Street, 700115 Iasi, Romania
[4] Department of Pharmacognosy, Faculty of Pharmacy, "Grigore T. Popa" University of Medicine and Pharmacy, 16 University Street, 700115 Iasi, Romania
[5] Center of Organic Chemistry "C.D. Nenitescu", Romanian Academy, Spl. Independentei 202B, 060023 Bucharest, Romania
* Correspondence: corneliamircea@yahoo.com (M.C.); acorciova@yahoo.com (A.C.)
† These authors contributed equally to this work.

Academic editors: Nazim Sekeroglu, Anake Kijjoa and Sevgi Gezici

Received: 30 July 2019; Accepted: 12 August 2019; Published: date

Abstract: *Zinnia elegans* (syn. *Zinnia violacea*) is a common ornamental plant of the Asteraceae family, widely cultivated for the impressive range of flower colors and persistent bloom. Given its uncomplicated cultivation and high adaptability to harsh landscape conditions, we investigated the potential use of *Z. elegans* as a source of valuable secondary metabolites. Preliminary classification of compounds found in a methanolic extract obtained from inflorescences of *Z. elegans* cv. Caroussel was accomplished using HR LC-MS techniques. The extract was then subjected to solid-phase extraction and separation using Sephadex LH-20 column chromatography, which resulted in several fractions further investigated for their antioxidant properties through lipoxygenase inhibition and metal chelating activity assays. Moreover, following additional purification procedures, structures of some active ingredients were established by NMR spectroscopy. The investigated fractions contained polyphenolic compounds such as chlorogenic acids and apigenin, kaempferol, and quercetin glycosides. Antioxidant assays showed that certain fractions exhibit moderate 15-LOX inhibition (Fr 2, IC_{50} = 18.98 μg/mL) and metal chelation (e.g., Fr 1-2, EC_{50} = 0.714-1.037 mg/mL) activities as compared to positive controls (20.25 μg/mL for kaempferol and 0.068 mg/mL for EDTA, respectively). For Fr 2, the 15-LOX inhibition activity seems to be related to the abundance of kaempferol glycosides. The NMR analyses revealed the presence of a kaempferol 3-*O*-glycoside, and a guanidine alkaloid previously not described in this species.

Keywords: *Zinnia elegans*; Asteraceae; guanidine alkaloids; HR-QTOF/MS; lipoxygenase; metal chelation.

Figure S1. ^{1}H NMR spectrum of compound **22** (500 MHz, MeOH-d_4, 30° C)

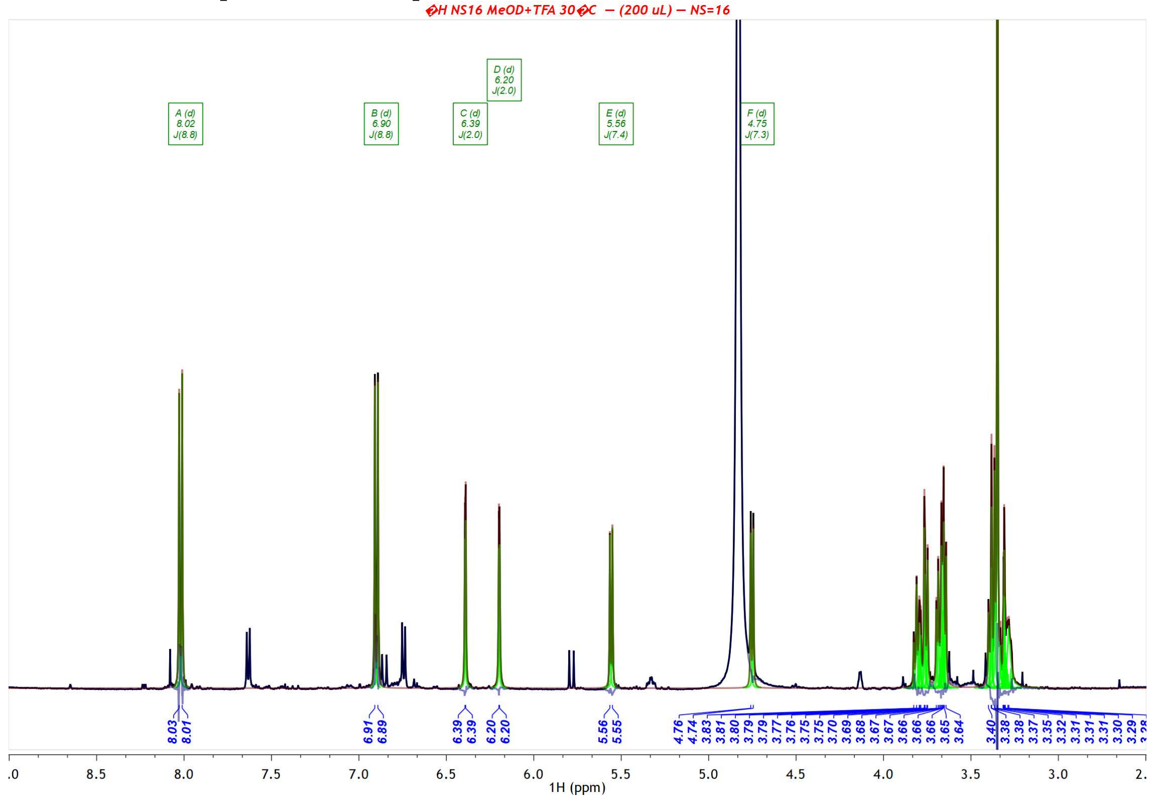

Figure S2. ^{13}C NMR spectrum of compound **22** (125 MHz, MeOH-d_4, 30° C)

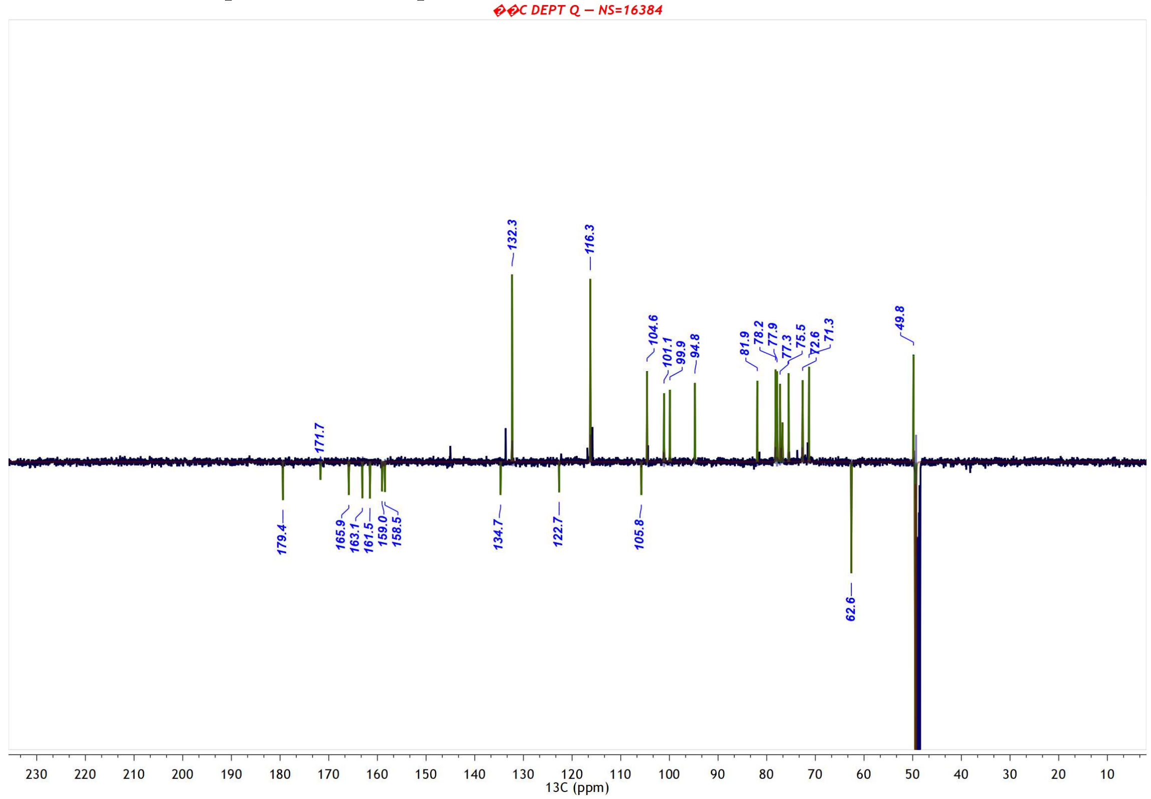

Figure S3. ^{1}H-^{1}H COSY NMR spectrum of compound **22** (500 MHz, MeOH-d_4, 30° C)

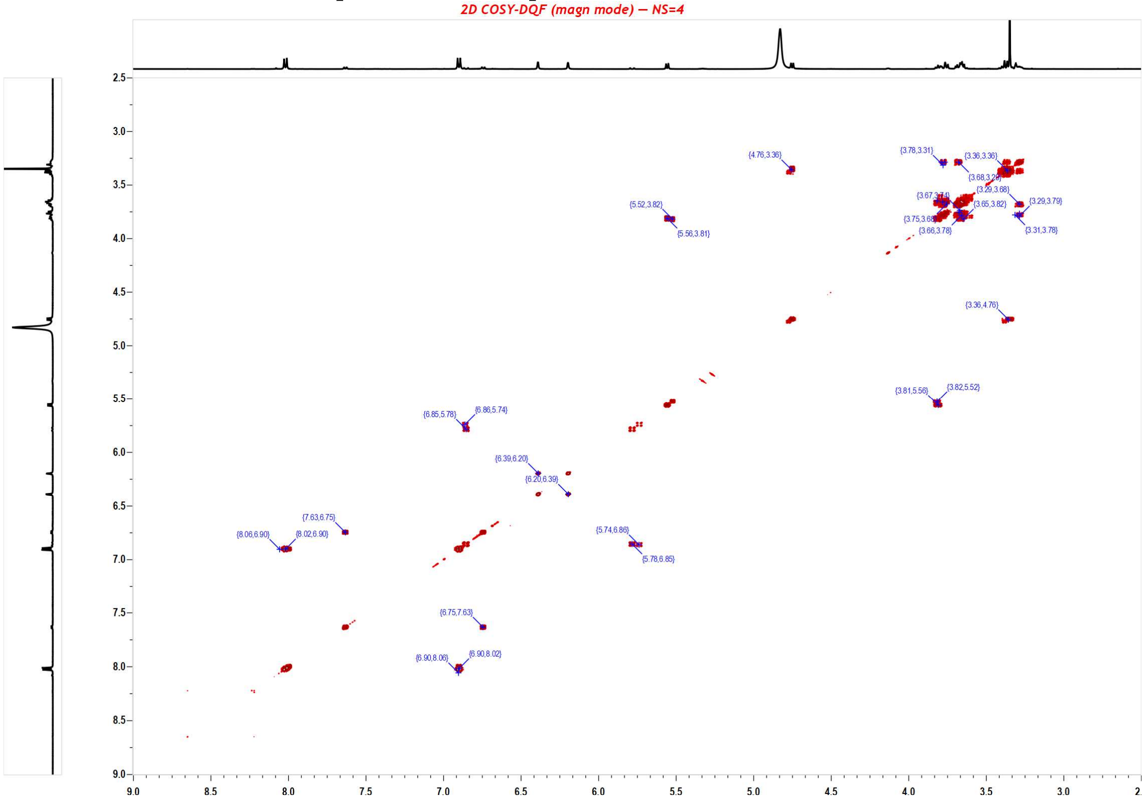

Figure S4. ^{1}H-^{1}H ROESY NMR spectrum of compound **22** (500 MHz, MeOH-d_4, 30° C)

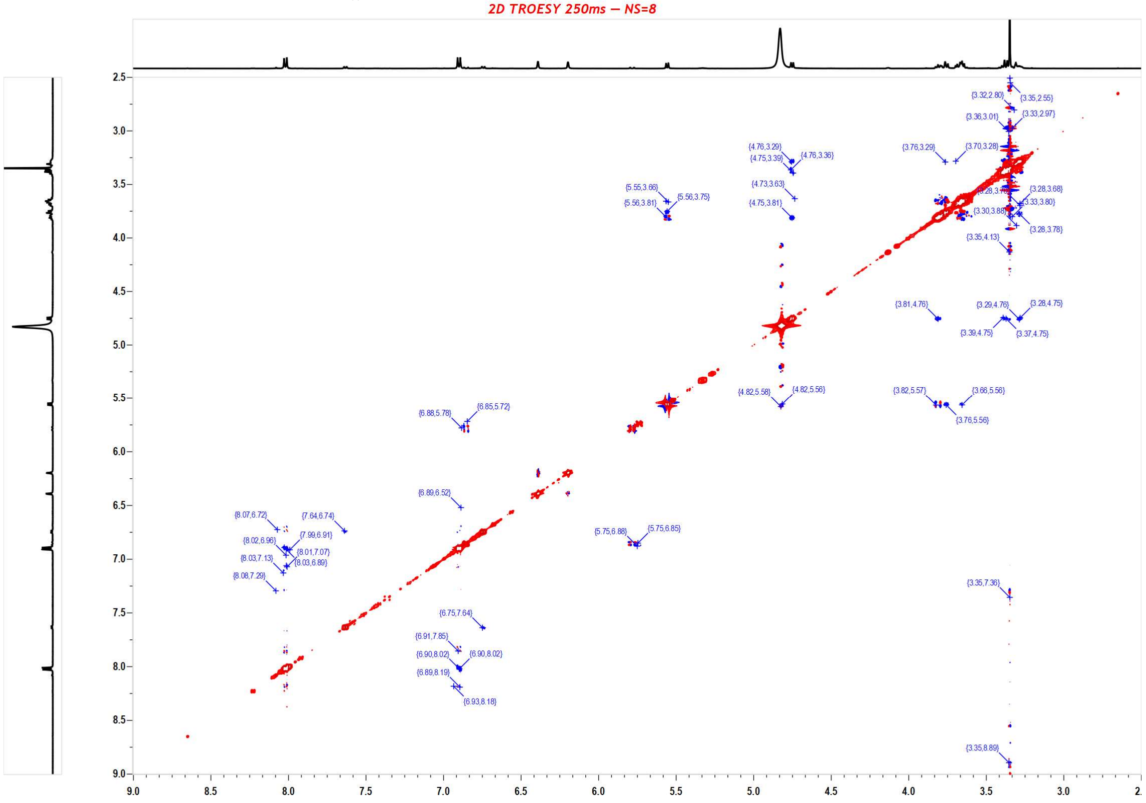

Figure S5. ^{1}H-^{13}C HSQC NMR spectrum of compound **22** (500/125 MHz, MeOH-d_4, 30° C)

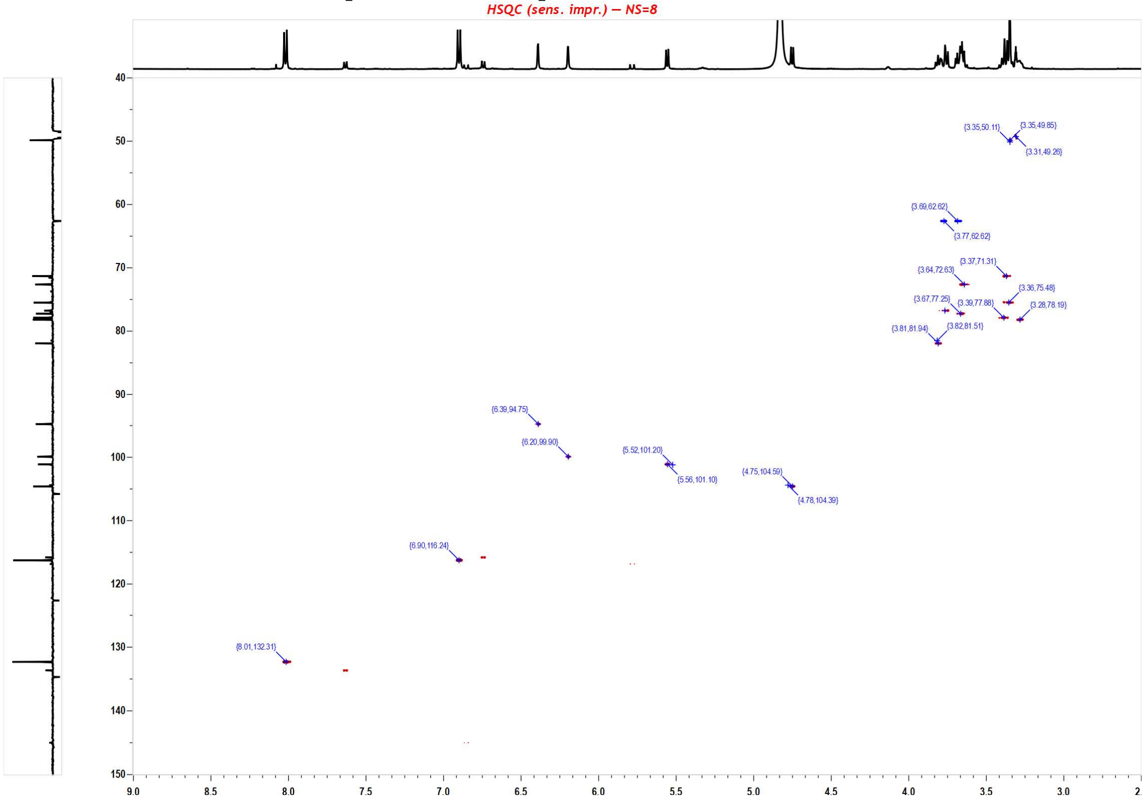

Figure S6. ^{1}H-^{13}C H2BC NMR spectrum of compound **22** (500/125 MHz, MeOH-d_4, 30° C)

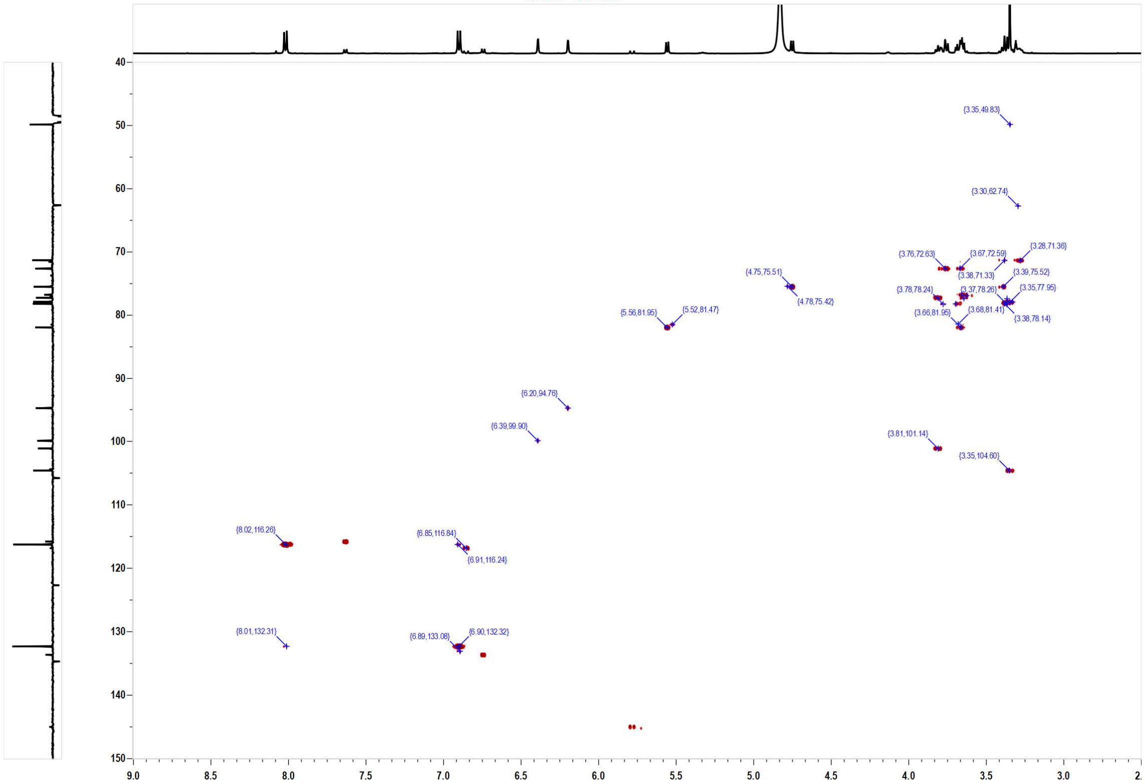

Figure S7. ^{1}H-^{13}C HMBC NMR spectrum of compound **22** (500/125 MHz, MeOH-d_4, 30° C)

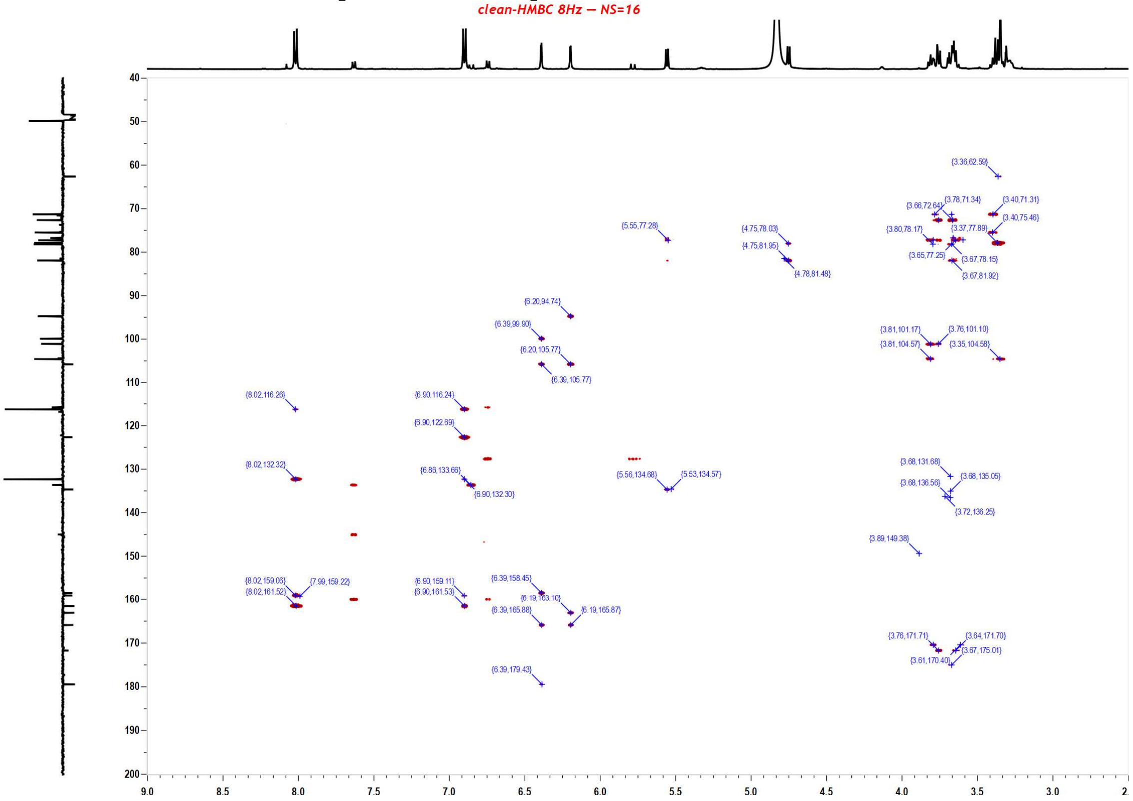

Supplement: Supplementary file 1 [file molecules-24-02934-s001.pdf]
